# Soluble polymorphic bank vole prion proteins induced by co-expression of quiescin sulfhydryl oxidase in *E. coli* and their aggregation behaviors

**DOI:** 10.1186/s12934-017-0782-x

**Published:** 2017-10-04

**Authors:** Romany Abskharon, Johnny Dang, Ameer Elfarash, Zerui Wang, Pingping Shen, Lewis S. Zou, Sedky Hassan, Fei Wang, Hisashi Fujioka, Jan Steyaert, Mentor Mulaj, Witold K. Surewicz, Joaquín Castilla, Alexandre Wohlkonig, Wen-Quan Zou

**Affiliations:** 1 VIB Center for Structural Biology, VIB, 1050 Brussels, Belgium; 2 Structural Biology Brussels, Vrije Universiteit Brussel (VUB), 1050 Brussels, Belgium; 3 National Institute of Oceanography and Fisheries (NIFO), Cairo, 11516 Egypt; 4 Departments of Pathology, Case Western Reserve University School of Medicine, Cleveland, OH USA; 5 Departments of Neurology, Case Western Reserve University School of Medicine, Cleveland, OH USA; 6 National Prion Disease Pathology Surveillance Center, Case Western Reserve University School of Medicine, Cleveland, OH USA; 7 National Center for Regenerative Medicine, Case Western Reserve University School of Medicine, Cleveland, OH USA; 8 The First Hospital of Jilin University, Changchun, Jilin Province People’s Republic of China; 9 State Key Laboratory for Infectious Disease Prevention and Control, National Institute for Viral Disease Control and Prevention, Chinese Center for Disease Control and Prevention, Beijing, People’s Republic of China; 10 Genetic Department, Faculty of Agriculture, Assiut University, Assuit, 71516 Egypt; 11 Center for Neurodegenerative Science, Van Andel Research Institute, Grand Rapids, MI 49503 USA; 12 Botany Department, Faculty of Science, Assiut University, New Valley Branch, El-Kharja, 72511 Egypt; 13 Electron Microscopy Core Facility, Case Western Reserve University School of Medicine, Cleveland, OH USA; 14 Department of Physiology and Biophysics, Case Western Reserve University School of Medicine, Cleveland, OH USA; 15 CIC bioGUNE, Parque Tecnológico de Bizkaia, 48160 Derio, Bizkaia Spain; 16 IKERBASQUE, Basque Foundation for Science, 48011 Bilbao, Bizkaia Spain

**Keywords:** Prions, Prion protein, Prion diseases, Quiescin sulfhydryl oxidase (QSOX), Bank vole, Thioflavin T (ThT), Surface plasmon resonance (SPR), Electron microscopy, Circular dichroism, Aggregation

## Abstract

**Background:**

The infectious prion protein (PrP^Sc^ or prion) is derived from its cellular form (PrP^C^) through a conformational transition in animal and human prion diseases. Studies have shown that the interspecies conversion of PrP^C^ to PrP^Sc^ is largely swayed by species barriers, which is mainly deciphered by the sequence and conformation of the proteins among species. However, the bank vole PrP^C^ (BVPrP) is highly susceptible to PrP^Sc^ from different species. Transgenic mice expressing BVPrP with the polymorphic isoleucine (109I) but methionine (109M) at residue 109 spontaneously develop prion disease.

**Results:**

To explore the mechanism underlying the unique susceptibility and convertibility, we generated soluble BVPrP by co-expression of BVPrP with Quiescin sulfhydryl oxidase (QSOX) in *Escherichia coli*. Interestingly, rBVPrP-109M and rBVPrP-109I exhibited distinct seeded aggregation pathways and aggregate morphologies upon seeding of mouse recombinant PrP fibrils, as monitored by thioflavin T fluorescence and electron microscopy. Moreover, they displayed different aggregation behaviors induced by seeding of hamster and mouse prion strains under real-time quaking-induced conversion.

**Conclusions:**

Our results suggest that QSOX facilitates the formation of soluble prion protein and provide further evidence that the polymorphism at residue 109 of QSOX-induced BVPrP may be a determinant in mediating its distinct convertibility and susceptibility.

## Background

Prion diseases are a group of fatal transmissible spongiform encephalopathies or neurodegenerative disorders that affect both humans and animals. Examples are Creutzfeldt–Jakob disease (CJD) and kuru in humans, scrapie in sheep and goats, bovine spongiform encephalopathy in cattle as well as chronic wasting disease in elk and deer. The typical spongiform degeneration, neuronal loss and astrocytosis characteristic of prion neuropathology are believed to be associated with the conversion of the detergent-soluble, α-helical rich monomeric cellular prion protein (PrP^C^) into the pathological detergent-insoluble, β-sheet rich, isoform (PrP^Sc^) [1]. Although it is known that all physicochemical changes are directly attributable to the structural transition, the molecular mechanism underlying the conversion from PrP^C^ into PrP^Sc^ remains poorly understood.

The structure of human PrP^C^ molecule has been characterized and it is composed of three helices (α1, α2, and α3) and two anti-parallel β-sheets (β1 and β2) folded into a characteristic β1-α1-β2-α2-α3 antiparallel beta-ribbon [2–7]. An intramolecular disulfide bridge (Cys 179–Cys 214) between α2 and α3 plays an important role in the folding and stability of PrP^C^ [2, 8, 9]. To fully understand the key molecular event in the pathogenesis of prion diseases, the structural conversion of PrP, generation of a soluble monomeric recombinant PrP^C^ in *Escherichia coli* that can be used for monitoring conformational conversion in vitro has been one of the important steps. However, expression of recombinant prion proteins (rPrP) in the cytoplasm of *E. coli* often forms inactive aggregates (termed inclusion bodies) [10]. These inclusion bodies must be solubilized in harsh reducing and denaturing conditions and then subsequently refolded in mild oxidizing conditions in order to restore the normal intramolecular disulfide bridge [11]. A consequence of the refolding process is some generation of incorrect intramolecular disulfide bridge between two molecules of the recombinant PrP. Furthermore, there is no enzymatic method to determine whether or not the correct folding occurs. Proper intramolecular folding can only be determined through additional characterization of the protein using circular dichroism (CD) or thioflavin T (ThT) fluorescence assays to validate the quality of the product [2].

Quiescin sulfhydryl oxidase (QSOX) is an enzyme that generates and transfers disulfide bond to protein substrates [12]. We have previously demonstrated the ability of QSOX to introduce a disulfide bond to the human and mouse prion proteins and also to facilitate the expression of soluble PrP in *E. coli* [13]. In this work, we described the production of soluble human and mouse prion proteins for the first time in the *E. coli* cytoplasm by the co-expression with QSOX [13]. Recently, we further observed that QSOX can highly and efficiently interact with PrP^Sc^, but not PrP^C^, isolated from the human brain by inhibiting PrP^Sc^ formation in vitro [14]. In the current study, we report that QSOX can be used to produce a large amount of soluble recombinant bank vole PrP (rBVPrP) with two different polymorphisms either rBVPrP-109I or rBVPrP-109M in *E. coli*. We also compare the aggregation behaviors of the two QSOX-induced rBVPrP molecules by amyloid fibril assay and electron microscope (EM). We investigated the ability of soluble rBVPrP-109I or rBVPrP-109M to be used as a substrate for hamster and mouse prion strains under real-time quaking-induced conversion (RT-QuIC). Bank voles have been demonstrated to be an animal model that has the least species barrier to other prion species and the rBVPrP has been reported to be a universal substrate for amplification of various prions by RT-QuIC assay [15, 16].

## Results

### QSOX-dependent expression of soluble BVPrP in *E. coli*

**Fig. 1:**
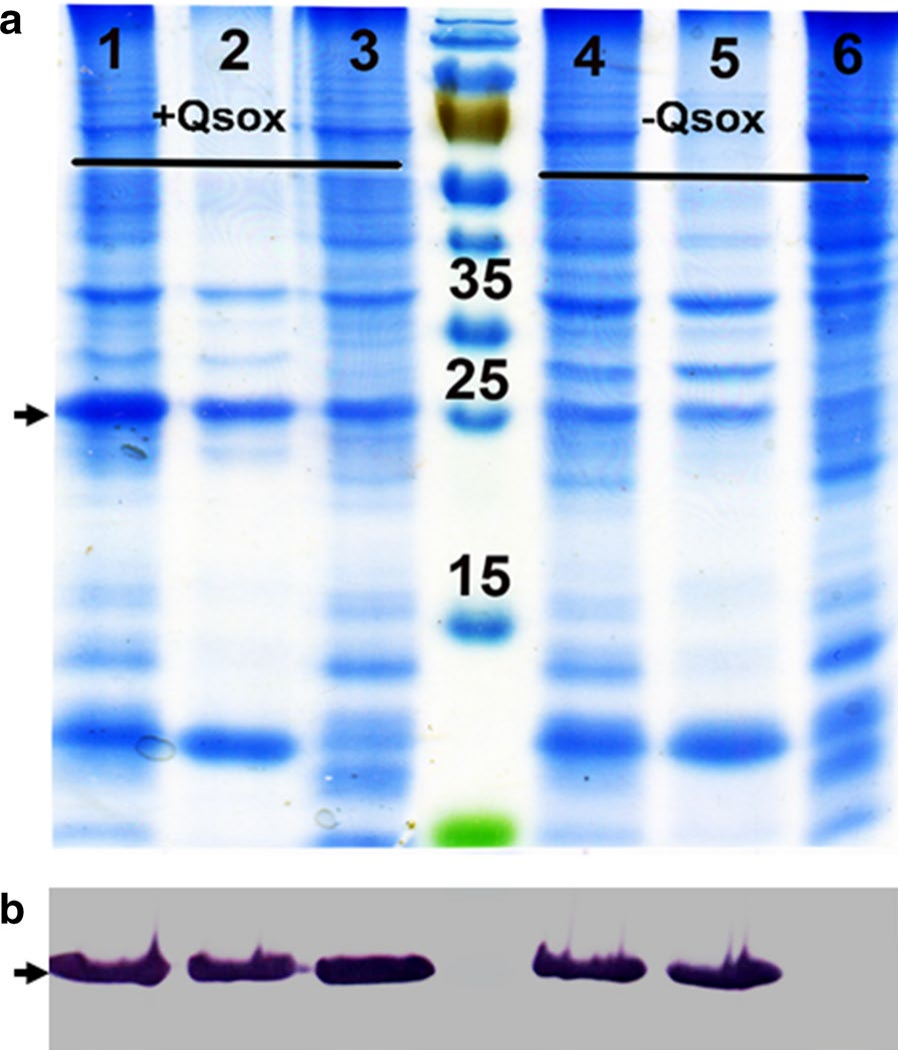
Comparison of the soluble over-expression of full-length Bank vole PrP 109M in the presence and absence of QSOX in *E. coli*. **a** Coomassie blue staining of SDS-PAGE gel (15%) shows the expression of rBVPrP in presence of QSOX: lanes 1–3 with QSOX: 1, total *E. coli* lysate; 2, insoluble fraction; 3, soluble fraction. Lanes 4–6 without QSOX: 4, total *E*. *coli* lysate; 5, insoluble fraction; 6, soluble fraction. **b** Western blotting of rBVPrP. The blot was probed with anti-His-tag antibody. The black arrows indicate the rBVPrP

To evaluate the effect of QSOX on expression of BVPrP-109M, *E. coli* Rossetta (DE3) pLysS containing the QSOX plasmid was transformed with pET28a-BVPrP-109M. A small-scale culture was grown to OD = 0.7, then induced with 1 mM IPTG at 15 °C for 16 h. Following lysis, the soluble and insoluble fractions were separated and analyzed on SDS-PAGE, then stained with Coomassie blue and Western blot probed in parallel with anti-His tag antibody (Fig. 1). In bacteria with QSOX, equivalent amounts of BVPrP were detected in both the soluble and insoluble fractions. Bacteria without QSOX, in contrast, exhibited detectable BVPrP-109M only in the insoluble fraction (Fig. 1). This result revealed that the generation of the soluble PrP is associated with the co-expression of QSOX in *E. coli*.

### Effect of QSOX on expression of rBVPrP in *E. coli* at different time points

**Fig. 2:**
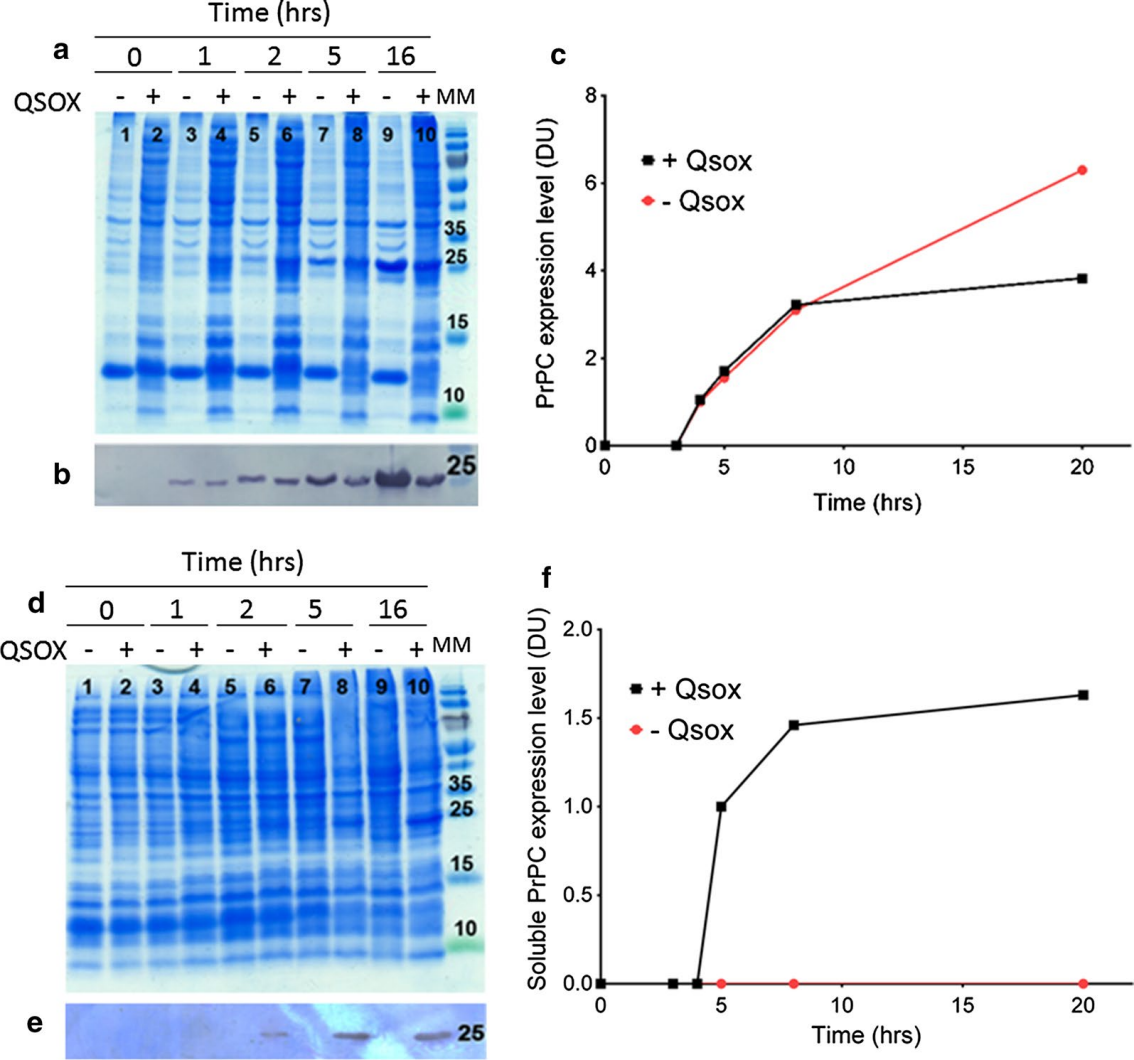
Production of rBVPrP-109M in the cytoplasm of *E. coli*. **a** Coomassie blue staining of the SDS-PAGE gel showing rBVPrP in the insoluble fraction. Lanes 1, 3, 5, 7, and 9 without QSOX; lanes 2, 4, 6, 8, and 10 with QSOX. Lanes 1 and 2, before induction; lanes 3 and 4, 1 h after induction; lanes 5 and 6, 2 h after induction; lanes 7 and 8, 5 h after induction; lanes 9 and 10, 16 h after induction; lane 11, molecular markers (MM). **b** Western blotting of rBVPrP in the insoluble fraction over time probed with the anti-His antibody. **c** Quantification of the protein in the insoluble fraction over time. **d** Coomassie blue staining of SDS-PAGE gel showing PrP in the soluble fraction over time as in **a**. **e** Western blotting of rBVPrP in the soluble fraction over time probed with the anti-His antibody. **f** Quantification of the expression of PrP in the soluble fraction over time

To further investigate the effect of QSOX on the production of rBVPrP, expression levels of soluble and insoluble BVPrP were monitored in the presence or absence of QSOX co-expression at different time points. SDS/PAGE and Western blot analysis showed that both cells co-expressed with and without QSOX posed equivalent insoluble fractions in the first 5 h after induction. However, upon a 16 h of induction, insoluble rBVPrP began to accumulate in cells without co-expression of QSOX (Fig. 2a–c). Only cells expressing QSOX produced soluble rBVPrP-109M, which was detectable after 2 h of induction (Fig. 2d, e). The level of the soluble BVPrP increased and reached a plateau after 8 h of induction (Fig. 2f).

### Purification of large amounts of rBVPrP by immobilized metal affinity chromatography and size exclusion chromatography

**Fig. 3:**
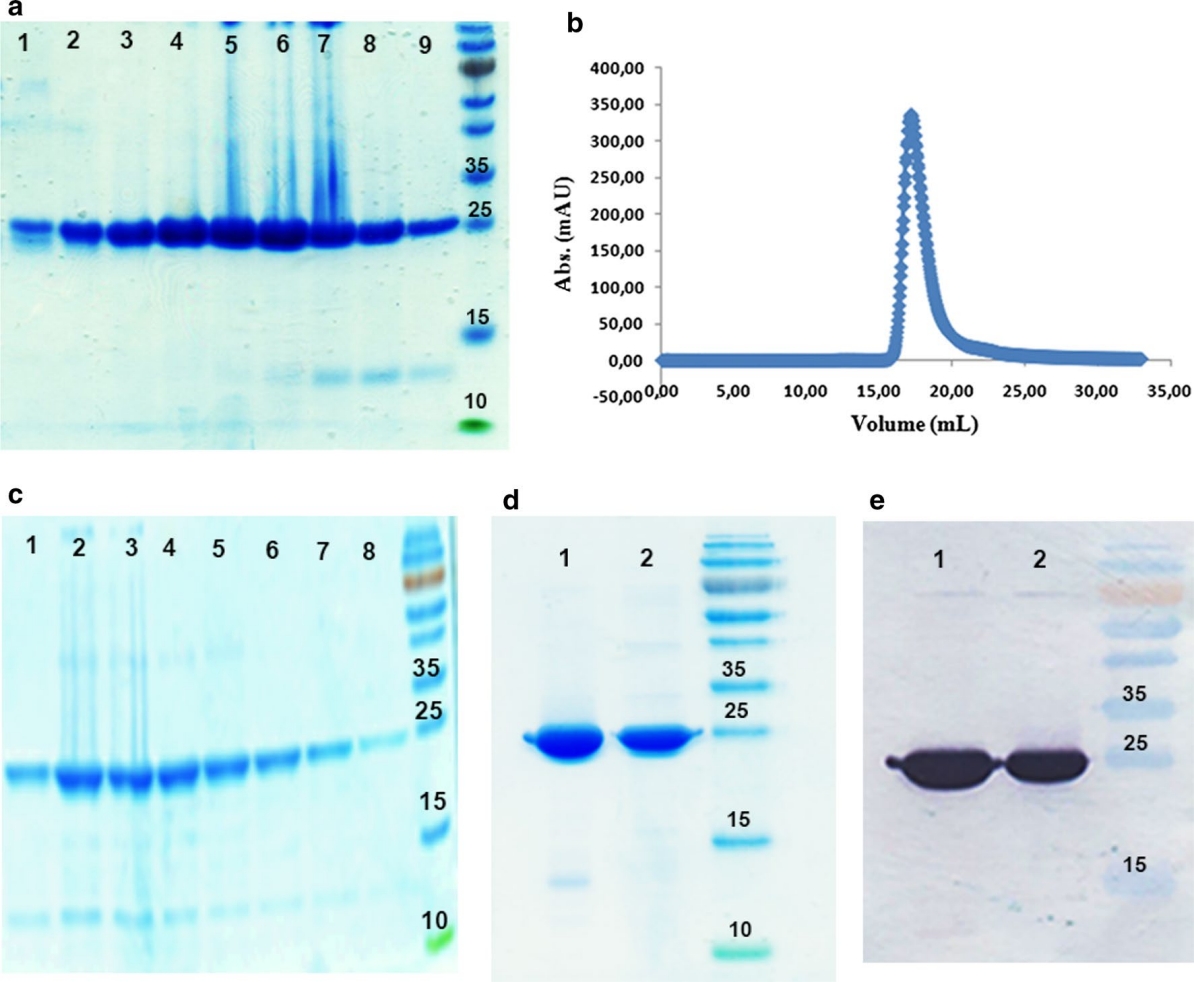
Production of soluble full-length bank vole PrP. **a** Coomassie blue staining of SDS-PAGE gel of full-length bank vole PrP 109M purified after Ni–NTA: lanes 1–9 are the elution peaks and lane 10 is the molecular marker. **b** Size exclusion chromatography (SEC) of full-length rBVPrP-109M using superdex 75 HR1030 column. **c** Coomassie blue staining of SDS-PAGE gel of purified full-length rBVPrP-109M after SEC. **d** Coomassie blue staining of the purified full-length bank vole PrP 109M and 109I after dialysis with 10 mM NaAC pH 4.6. **e** Western blotting of purified rBVPrP probed with the anti-His-tag antibody as in **d**

To generate a large amount of purified protein for structural and functional studies, a liter of *E. coli* culture was induced for 16 h at 15 °C. The soluble fraction of full-length rBVPrP-109M was then subjected to immobilized metal affinity chromatography (IMAC) (Fig. 3a), followed by size exclusion chromatography (SEC) in order to maximize purity (Fig. 3b, c). The eluted fractions from SEC were tested by SDS-PAGE and Western blotting. The same procedure was used to purify rBVPrP-109I, but with a yield that was tenfold lower than that for rBVPrP-109M (1 mg/L vs. 10 mg/L per liter of culture) (Fig. 3d, e).

### Circular dichroism spectroscopy

**Fig. 4:**
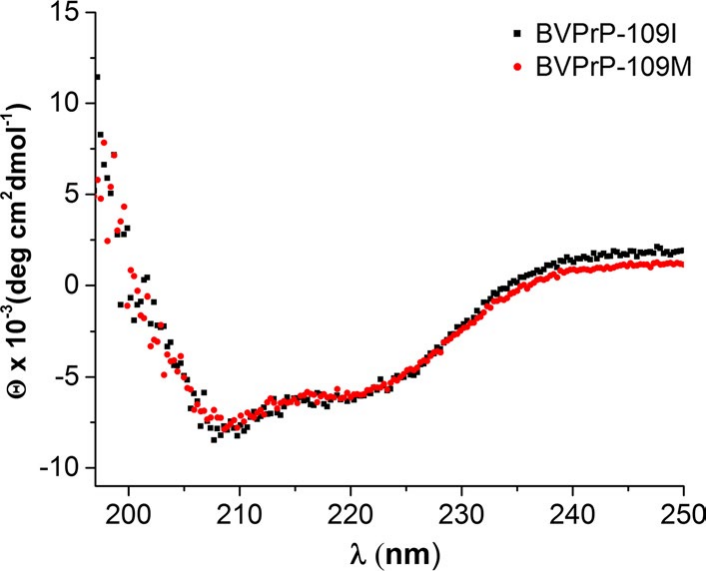
Secondary structural features of bank vole prion proteins. Far-UV circular dichroism spectra of soluble rBVPrP-109M and rBVPrP-109I are shown in red and black, respectively. All spectra were recorded at 25 °C in 20 mM sodium citrate buffer pH 5

We used UV circular dichroism (CD) spectroscopy to confirm that the secondary structure of both purified rBVPrP-109M and rBVPrP-109I was primarily α-helical in structure. The CD spectra of both proteins are similar to those reported for prion proteins from other species [17, 18], exhibiting double minimum at 208 and 222 nm characteristic of α-helical secondary structure (Fig. 4). These results confirmed that the purified BVPrP molecules were correctly folded.

### Interaction of QSOX with BVPrP proteins

**Fig. 5:**
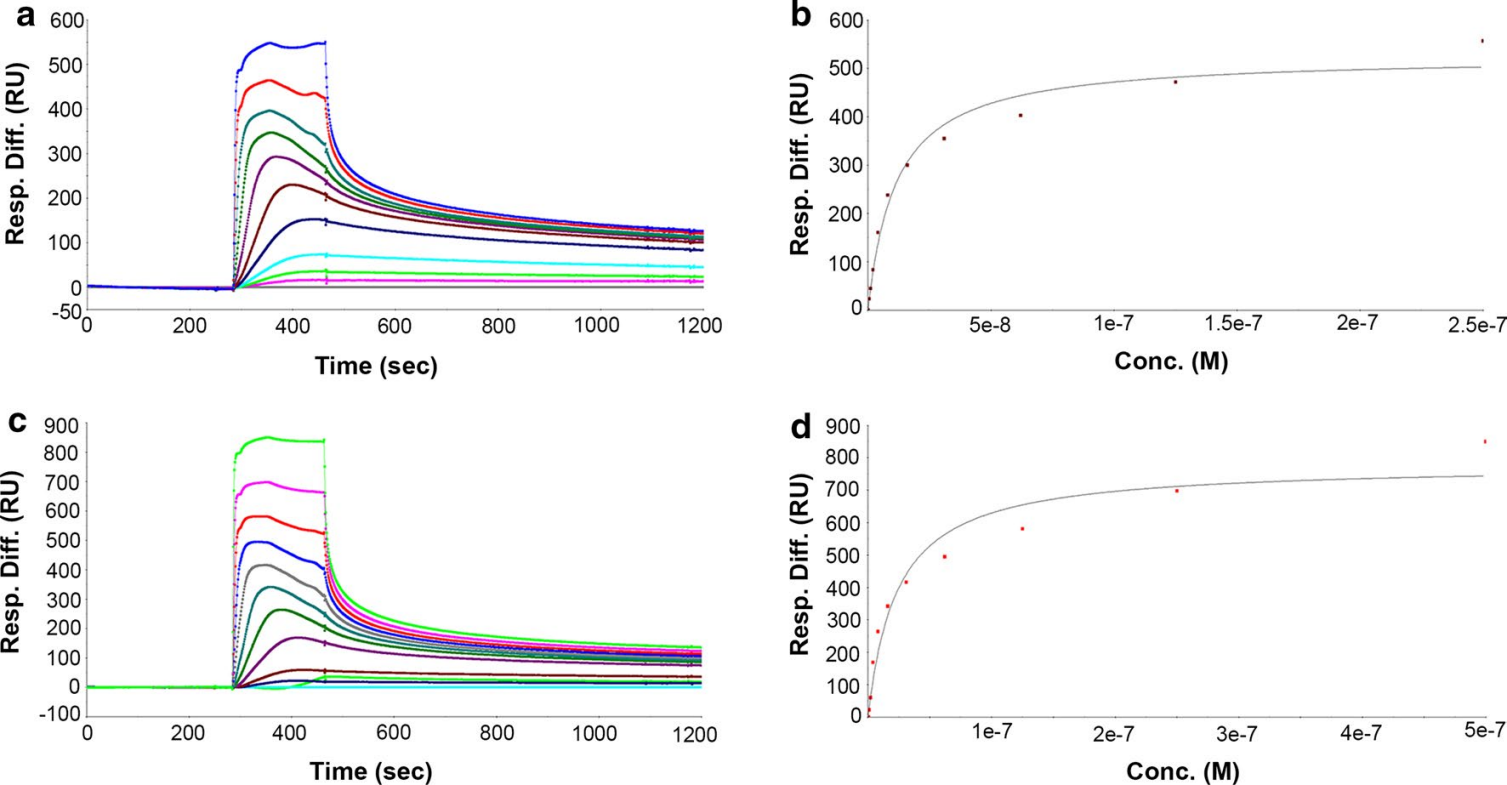
Detection of the interaction of rBVPrP with QSOX. **a**, **c** Binding spectra of the full-length rBVPrP-109M and rBVPrP-109I with QSOX, respectively. **b**, **d** Responses plotted vs bank vole PrP 109I and 109M concentrations, respectively. The interaction is fitted to 1:1 Langmuir model

Our recent study demonstrated that QSOX is able to bind different species of PrP^Sc^ and inhibit PrP^Sc^ formation in vitro [14]. Surface Plasmon Resonance (SPR) was used to determine the dissociation constant (*Kd*) of this interaction. QSOX was immobilized on the surface of a CM5 chip and full-length rBVPrP-109M or rBVPrP-109I was analyzed at various concentrations to determine binding kinetics. The dissociation constant was determined from the change of the refractive index following the interaction of QSOX with rBVPrP. The *Kd* values for full-length BVPrP-109M was 23 and 11 nM for BVPrP-109I, respectively (Fig. 5).

### Aggregation of rBVPrP

**Fig. 6:**
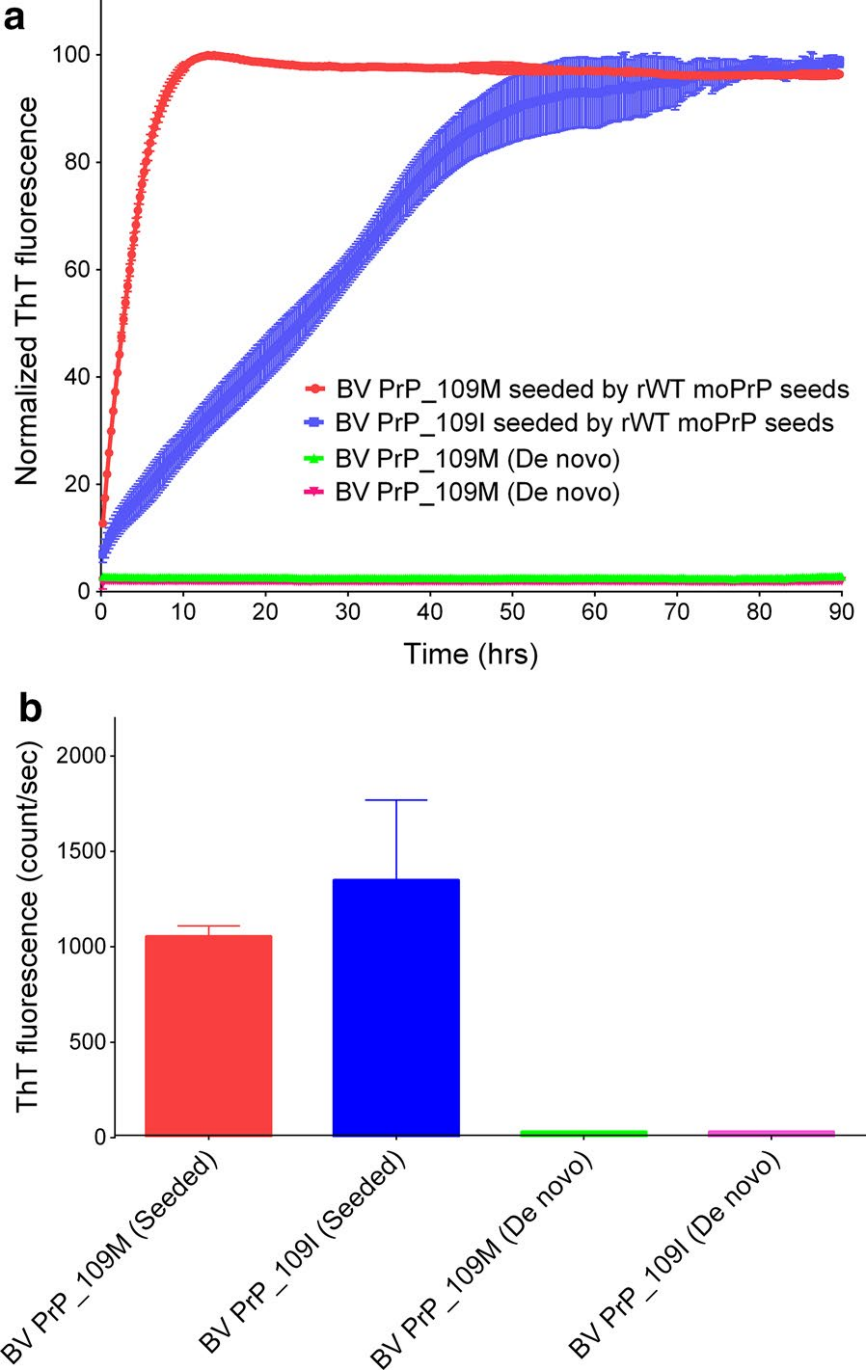
Aggregation kinetics of rBVPrP-109M and rBVPrP-109I. **a** ThT kinetics of fibril formation for rBVPrP-109M and rBVPrP-109I seeded by rMoPrP (23–230) seeds and incubated in 2 M GdnHCl, 100 mM potassium phosphate buffer pH 6.5, 20 μM ThT. **b** Mean value of the maximum ThT intensity (n = 4)

**Fig. 7:**
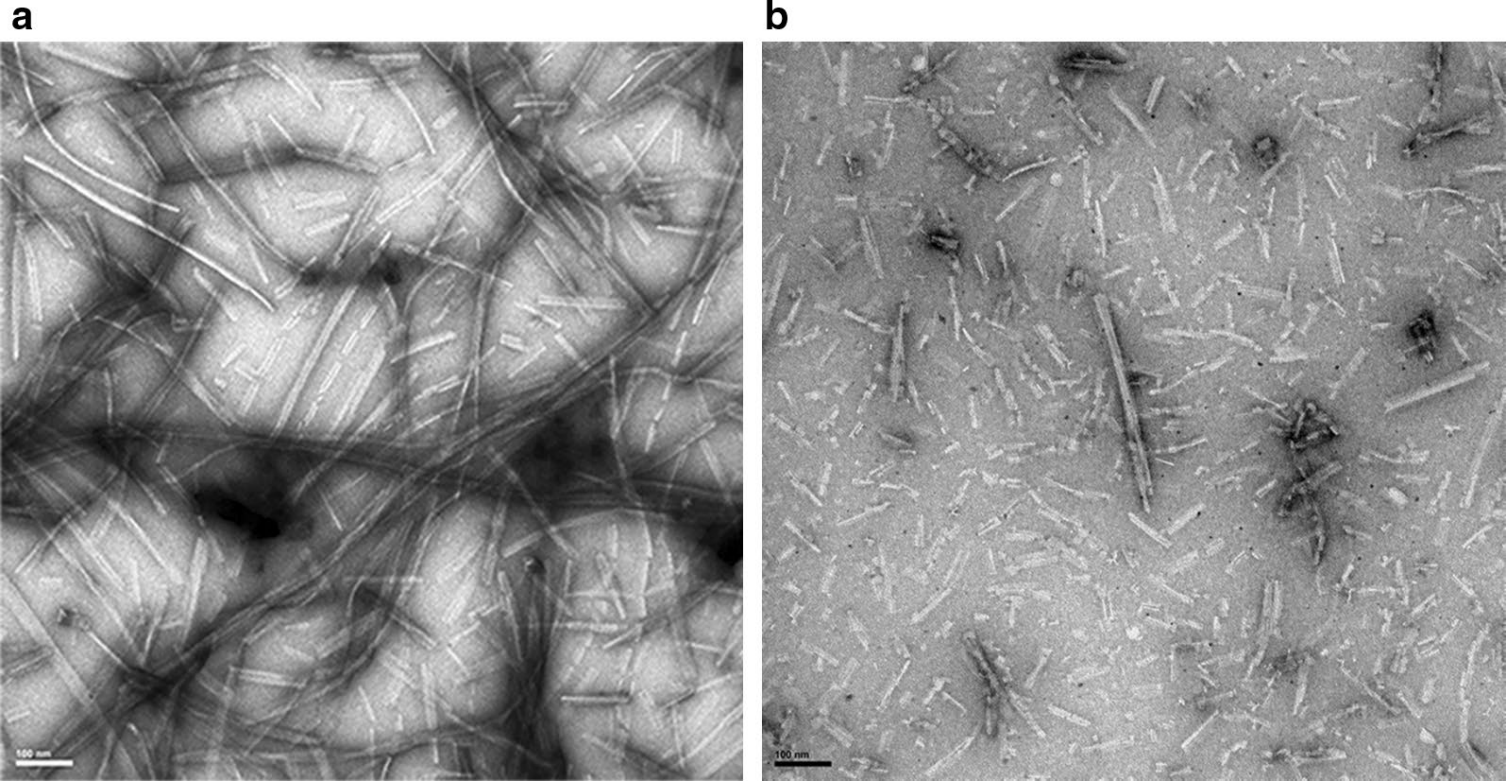
Electron microscopy images of rBVPrP fibrils. **a** Bank vole PrP109M. **b** Bank vole PrP109I. The scale bar 100 nm

To determine the aggregation kinetics of rBVPrP-109M or rBVPrP-109I, we used thioflavin T (ThT) as a fluorescence probing dye to monitor protein aggregation. ThT has been widely used to determine protein aggregation and the fluorescence increases upon the binding of ThT to β-rich amyloid like structure. In the seeded reaction using wild-type recombinant mouse PrP fibrils, both recombinant bank vole PrP molecules were able to form amyloid fibrils with virtually no lag phases, through with different kinetics (Fig. 6a). BVPrP-109M more rapidly reached the plateau than BVPrP-109I. In unseeded reactions, neither soluble recombinant bank vole PrP proteins were able to form de novo fibrils after 90 h (Fig. 6a). These data suggest that soluble rBVPrP-109M and rBVPrP-109I can be seeded by aggregates from different species to form ThT-positive fibrils (Fig. 6). To gain further insights into the aggregated morphology, we used electron microscope (EM) to visualize the structure of rBVPrP aggregates. EM images confirmed the formation of long mature fibrils with 100 nm scale bars (Fig. 7). Interestingly, more mature PrP fibrils were observed in rBVPrP-109M than in rBVPrP-109I, which may be associated with their distinct aggregation pathways in the seeded reaction.

### Recombinant BVPrP serving as substrates in RT-QuIC assay

**Fig. 8:**
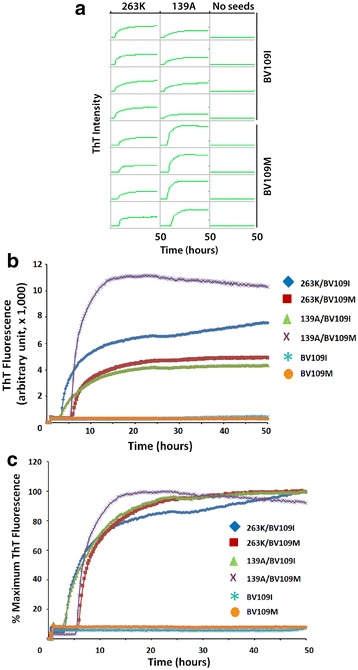
rBVPrP-109M and rBVPrP-109I used as substrates for RT-QuIC analysis of hamster 263 K and mouse139A. **a** RT-QuIC spectra of 263 K and 139A in the presence of rBVPrP109I or rBVPrP109M as a substrate, respectively. 2 µL of brain homogenate diluted at 10^−3^ from either 263 K-infected hamster brain or 139A-infected mouse brain was added into each well of the 96-well plates as seeds. Each well contained 98 µL RT-QuIC reaction solution [10 mM phosphate buffer at pH 7.4, 300 mM NaCl, 10 µM thioflavin T (ThT), 1 mM EDTA, and 0.1 mg/mL of either rBVPrP109I (top four rows) or rBVPrP109M (bottom four rows)]. Negative controls were the samples without PrP^Sc^ seeds. **b** Comparison of ThT fluorescence intensity of RT-QuIC prion seeding activity of rBVPrP with 109I or 109M polymorphism seeded by 263 K or 193A strains. The ThT fluorescence intensity is plotted as a function of reaction time (hours). **c** Comparison of the lag phase of the RT-QuIC seeding activity of rBVPrP with 109I or 109M polymorphism seeded by 263 K and 193A stains. Percentage of maximal ThT fluorescence is plotted as a function of reaction time (hours)

Recent studies have revealed that BVPrP is highly convertible to PrP^Sc^ or aggregates by prions from a variety of species in vivo and in vitro [15, 16]. To determine whether QSOX-induced soluble rBVPrP carrying either 109M or 109I can be used as a substrate in real-time quaking-induced conversion (RT-QuIC), we conducted RT-QuIC analysis of hamster-adapted scrapie prion strain 263 K and mouse-adapted scrapie prion strain 139A as previously described [16]. Both rBVPrP forms can be induced into PrP aggregates by either 263 K or 193A prion strain via RT-QuIC assay (Fig. 8). For hamster 263 K strain, rBVPrP-109I exhibited a near twofold greater prion-seeding activity compared to rBVPrP-109M; in contrast, rBVPrP-109I had almost a threefold lower prion-seeding activity compared to rBVPrP-109M when with mouse 193A strain (Fig. 8a, b). Nevertheless, rBVPrP-109I revealed a much more rapid seeding activity than rBVPrP-109M with both 263 K and 139A strains (Fig. 8a, c).

## Discussion

Bacteria are simple and cost effective systems for producing recombinant proteins. However, the over-expression of recombinant proteins in bacteria often generates misfolded proteins, which form inclusion bodies in the cytoplasm [10]. As more applications require higher amounts of high-quality recombinant proteins, new systems and refinements to recombinant protein expression have been developed, notably simultaneous over-expression of chaperones for proper folding and solubility of generated proteins during expression [19]. For example, an established strategy to overcome the formation of inclusion bodies is to introduce a chaperone that helps solubilize the recombinant proteins [19]. Different proteins have been observed to interact with prion protein [20], namely, molecular chaperones from the endoplasmic reticulum (ER), such as Pdia3, Grp58, and Hsp60 [20–22]. This is suggestive of an important role for molecular chaperones in early protein folding. Most of these chaperones possess protein disulfide isomerase-like properties or folding activities [23]. In the ER, disulfide bond formation is catalyzed by Ero1 and the PDI [24, 25]. The over-expression of the molecular chaperones (GroELS, Skp or trigger factor) and isomerase (DsbC) has been shown to significantly increase the production yield of recombinant disulfide bond-containing antibodies [26]. Indeed, we have previously observed that co-expression of the chaperone QSOX prevents human or mouse PrP from aggregation by producing soluble PrP in *E. coli* [13].

In bacteria, correct oxidative protein folding depends on both the disulfide bond protein A and B (DsbA/DsbB) pathway, which catalyzes disulfide bond formation and disulfide bond isomerization [11]. In eukaryotes, ER oxidoreductin 1 (Ero1) and the protein disulphide isomerase (PDI) catalyze the equivalent reactions in the ER. Surprisingly, QSOX has been found to be the only known enzyme that is able to implement both reactions to generate and transfer the disulfide bridge for protein substrates [12]. Because proper folding and normal physicochemical properties of proteins produced in *E. coli* rely on formation of disulfide bonds, it is conceivable that co-expression of QSOX may facilitate generation of soluble properly folded PrP. Our current study with co-expression of BVPrP and QSOX in *E. coli* further showed soluble BVPrP, confirming our previous study. Additionally, the CD spectra of the rBVPrP proteins are in agreement with the NMR and X-rays structures of the soluble rBVPrP containing mostly α-helical structures [5, 11].

Bank voles (*Myodes glareolus*) have been well-demonstrated to be an important animal model for prion research because it is highly susceptible to a wide range of prion strains including human, cattle, elk, sheep, mice and hamsters [15]. Compared to other animal models, such as mice, hamsters, or humanized transgenic (Tg) mice, prions from several species are transmissible to bank voles with a higher attack rate and shorter incubation time [27–30]. The molecular mechanism underlying this phenomenon remains unknown. It is conceivable that this highly efficient susceptibility is attributable to the sequence and structure of BVPrP. Consistent with this hypothesis, interestingly, rBVPrP-109M has been found to be a universal substrate for determining seeding activity of prions from a diverse range of species by real-time quacking-induced conversion (RT-QuIC) assay in vitro [16].

BVPrP contains a polymorphism at residue 109, with either methionine (M) or isoleucine (I) [27, 31]. Notably, the transmission of CWD to Tg mice expressing BVPrP-109M exhibited a longer incubation time than Tg mice carrying BVPrP-109I [31]. We observed that *E. coli* containing QSOX and BVPrP-109M plasmids expressed soluble PrP 10 times more than the one transformed with QSOX and BVPrP-109I. It would be interesting to know whether the distinct susceptibility between BVPrP-109I and BVPrP-109M is related to a different expression level in both strains. Watts et al. have reported that Tg mice expressing BVPrP-109I spontaneously developed prion diseases, whereas Tg mice expressing BVPrP-109M did not [27]. It is possible that this polymorphism may affect the expression level or structure of the protein as human PrP polymorphisms do [32–34].

## Conclusion

We successfully produced soluble rBVPrP-109M or rBVPrP-109I with the co-expression of QSOX in cytoplasm of *E. coli*. Interestingly, we found that rBVPrP-109M and rBVPrP-109I experienced different aggregation kinetics, with the former reaching the plateau quicker than the latter in the presence of recombinant mouse PrP aggregate seeds. Moreover, there was a difference in morphology of aggregates—rBVPrP-109M formed mature fibrils, whereas rBVPrP-109I mainly generated shorter protofibril-like morphology. With the RT-QuIC system, we revealed that the two rBVPrP responded differently to the infectious hamster and mouse prion strains. Whether their distinct aggregation behaviors or different responses to seeding are associated with different structural features remains to be further determined.

## Methods

### Ethics approval and consent to participate

All experiments conducted in this study were monitored and approved by VIB Center for Structural Biology, Brussels, Belgium and Case Western Reserve University School of Medicine, Cleveland, Ohio, USA.

### Cloning of BVPrP into expression construct

The DNA coding for full-length BVPrP-109M and BVPrP-109I was amplified by PCR using a template plasmid of BVPrP-109M/pOPINE or BVPrP-109I/pOPINE. The amplification was carried out using oligonucleotides 5′ CGCGGATCCATGAAGAAGCGGCCAAAGCCTGG 3′ and 5′ CCCAAGCTTTTAGGAACTTCTCCCTTCGT 3′. The PCR product was digested with *Bam*HI and *Hin*dIII, and inserted into pET-28a (Novagen).

### Small-scale expression of BVPrP in *E. coli*

To avoid formation of inclusion bodies and increase the yield of soluble full-length BVPrP, we co-transformed *E. coli* Rossetta (DE3) pLysS with each BVPrP construct and the QSOX plasmid as previously described [13, 35]. The transformed cells were plated on LB-agar supplemented with 100 μg/mL ampicillin and 50 μg/mL kanamycin. Fresh transformed cells were used to inoculate a 10 mL pre-culture (LB medium supplemented with above antibiotics). The next day, a 40 mL culture was inoculated at 37 °C with 1 mL of the pre-culture and induced with 1 mM isopropyl-b-d-thiogalactopyranoside (IPTG) at an optical density (OD_600_) of 0.7. After induction, the culture temperature was shifted to 15 °C and incubated overnight (16 h). Cells were pelleted by centrifugation and resuspended in ice cold lysis buffer: 0.1*g* of cell paste/mL of 50 mM potassium phosphate, pH 7.5, 300 mM NaCl supplemented with 0.1 mg/mL lysozyme, 0.1 mg/mL ABESF and 1 μg/mL leupeptin. Cells were lysed by sonication for 4 times, each time 30 s at 4 °C and were subsequently centrifuged for 20 min at 18,000*g*. The supernatant was collected and the pellet was resuspended in the initial volume using lysis buffer. To analyze the expression of the soluble BVPrP, SDS/PAGE and immunoblotting were performed for total, supernatant, and pellet fractions as previously described [13].

### Quantification of BVPrP expression in *E. coli*

To quantify the amount of BVPrP produced at the different time points during the growth, 1 L culture was induced as described earlier and a 40 mL of sample was collected at 0, 1, 2, 4 and 16 h after induction.

Cells were collected by centrifugation at 15,000*g* for 10 min, weighted and resuspended in (0.1 g of cell paste/mL) volume of lysis buffer to normalize the cell content for each time point. For estimating the production of total prion protein, a 4 μL of lysis was mixed with 1 μL SDS loading buffer (5 ×) and boiled for 5 min prior to loading onto gels for Western blotting. To determine the amount of total soluble proteins expressed, resuspended cells were lysed by sonication and centrifuged at 18,000*g* for 20 min. A 4 μL of supernatant was mixed with 1 μL SDS loading buffer (5 ×) and boiled for 5 min prior to loading to gels for staining with Coomassie blue. Collected samples were analyzed on SDS-PAGE and by immunoblotting probed with monoclonal anti-His antibody (Sigma Aldrich) on nitrocellulose membranes (MACHEREY–NAGEL). The PrP bands were visualized by goat anti-mouse IgG, alkaline phosphatase conjugate (Sigma) using NBT/BCIP as substrate (Roche Diagnostics, GmbH). Intensity of the blot signals was quantified using the software LabImage 1D Gel Analysis (Kapelan GmbH, Germany).

### Large-scale protein expression and purification

The *E. coli* Rossetta (DE3) pLysS were initially co-transformed with full-length BVPrP-109M or BVPrP-109I plus QSOX for a large scale production. *E. coli* pre-cultures cells (25 mL) were grown overnight at 37 °C in LB medium with supplemented ampicillin (100 μg/mL) and kanamycin (50 μg/mL). A 10 mL of pre-culture was used to inoculate 1 L of LB medium supplemented with ampicillin and kanamycin. The bacteria were induced at A_600_ = 0.6 by adding 1 mM isopropyl-b-d-thiogalactopyranoside (IPTG) and then subsequently grown at 15 °C for 16 h. Cells were collected by centrifugation (15 min at 15,000*g*). The bacterial pellets were resuspended as 0.1 g of cell paste/mL in lysing buffer (50 mM potassium phosphate, pH 7.5, 300 mM NaCl supplemented with 0.1 mg/mL lysozyme, 0.1 mg/mL AEBSF and 1 μg/mL leupeptin). Mechanical disruption was used to lyse the cells using a French press (10,000 psi) and followed by centrifugation at 4 °C for 60 min at 40,000*g*. The collected supernatant was loaded on a 5 mL Histrap Ni–NTA column (GE-healthcare) previously equilibrated with equilibration buffer (50 mM potassium phosphate pH 7.5, 300 mM NaCl, 10 mM imidazole). The column was washed with five column volumes (CV) of washing buffer: 50 mM potassium phosphate pH 7.5, 1 M NaCl, 50 mM imidazole, followed by ten CV volumes of 50 mM potassium phosphate pH 6.0, 1 M NaCl, and 50 mM imidazole. The protein was eluted with a gradient of imidazole from 50 mM to 1 M in 50 mM potassium phosphate pH 7.5.

The eluted soluble BVPrP fractions were loaded on a SDS/PAGE to evaluate protein purity and then pooled and concentrated for a second purification step. The concentrated pool was applied onto a Superdex 75 HR 10/300 GL (GE Healthcare) and eluted with 20 mM Tris–HCl pH 7.5 containing 150 mM NaCl. The elution peak fractions were loaded on SDS/PAGE. The fractions only containing BVPrP were collected and dialyzed against 10 mM sodium acetate pH 4.6 and 1 mM EDTA followed by the final dialysis buffer of 10 mM sodium acetate pH 4.6. Protein aliquots were stored at − 80 °C until further usage.

### Circular dichroism spectroscopy

The far-UV circular dichroism (CD) spectra of rBVPrP were recorded on a Aviv 215 spectropolarimeter (Tokyo, Japan) as previously described [36]. The measurements were performed in 20 mM sodium citrate buffer (pH 5) at 25 °C using a 1-cm path length cell. Protein concentration used to normalize the spectra was determined using a molar extinction coefficient at 280 nm of 62,005/M/cm.

### Aggregation of rBVPrP monitored by thioflavin T fluorescence assay

To monitor the amyloid fibrils formation of QSOX-induced rBVPrP, the thioflavin T (ThT) fluorescence assay was used. We first used 0.5 mg/mL of rBVPrP-109M incubated in 2 M GdnHCl, 100 mM potassium phosphate buffer pH 6.5, and 20 μM ThT. The reaction volume was 200 μL per well in 96-well plates (Corning). Seeding was achieved by adding 1 μL of seeds (0.5 mg/mL), to each well in 96-well plates. The seeds were prepared previously from recombinant moPrP23–230 fibrils in the same buffer conditions [37]. The 96-well plate was incubated at 37 °C with continuous shaking on a plate reader (SYNERGY2, BioTek). The fibril kinetics was monitored by measuring ThT fluorescence intensity every 15 min by using 440-nm excitation and 480-nm emission. The amyloid-formation kinetics were calculated from four replicates.

### Electron microscopy of rBVPrP-109M and rBVPrP-109I

Transmission electron microscopy of rBVPrP was performed as previously described [38]. Formvar/carbon coated EM nickel grids (400 mesh) were placed formvar/carbon side down on top of a drop of the amyloid fibrils solution (0.5 mg/mL) for 1 min. The grids were removed, blotted with filter paper and placed onto a drop of 2.0% uranyl acetate (UA) solution for 1 min. The excess UA was removed, and the EM grids were air-dried. The grids were observed by an FEI Tecnai Spirit (T12) electron microscope and the images were captured by a Gatan US4000 4k × 4k CCD camera.

### Surface plasmon resonance experiments

The interaction between QSOX and the full-length rBVPrP-109M or rBVPrP-109I was studied using surface plasmon resonance (SPR) with a BIAcore 3000 instrument as described previously [14]. QSOX was diluted to 2 µg/mL in 10 mM sodium acetate, pH 5.2, and covalently linked to a Sensor Chip CM5 (carboxymethylated dextran surface) using the amine coupling chemistry. A surface density of 1500 RU was created after immobilization and blocking with ethanolamine. Different concentrations of the full-length rBVPrP-109M or rBVPrP-109I (0–500 nM) were injected in a running buffer (PBS, pH 7.4, 0.005% surfactant P20 and 3 mM EDTA) at 25 °C at a flow rate of 5 µL/min. All analytes were run subsequently over a control flow cell containing a blank surface (with no immobilized protein). After each cycle, the surface was regeneration with 60 s pulse of 100 mM glycine, pH 1.5. Association rates (*K*_*on*_) and dissociation rates (*K*_*off*_) were obtained using a 1:1 Langmuir binding model (Bicore evaluation software version 4.1). The equilibrium dissociation constant (*K*_*d*_) was calculated using steady state fitting.

### RT-QuIC assay

RT-QuIC assay was conducted as previously described [16]. The seeds used in this study were prepared as 10% (w/v) brain homogenates from hamster-adapted scrapie strain 263 K and mouse-adapted scrapie strain 139A. They were diluted 1000-fold in a solution containing 0.1% sodium dodecyl sulfate (SDS), 1X phosphate buffered saline at pH 5.8, and 1X N2 media supplement. The RT-QuIC reaction solution was prepared to contain 10 mM phosphate buffer at pH 7.4, 300 mM NaCl, 10 µM thioflavin T (ThT), 1 mM ethylenediaminetetraacetic acid (EDTA), and 0.1 mg/mL of either rBVPrP23–231 (109M) or rBVPrP23–231 (109I). 98 µL of reaction solution was loaded into a black clear-bottomed 96 well plate (Nunc). Wells were subsequently seeded with 2 µL of either hamster 263 K or mouse 139A diluted brain homogenate for a final reaction of 100 µL with a final SDS concentration of 0.002%. Plates were then sealed and placed in a BMG FLUOstar Omega plate reader at 55 °C for 50 h and subjected to 60 s intervals of double orbital shaking at 700 rpm, alternating with 60 s intervals of rest. ThT fluorescence measurements, excitation at 450 nm and emission at 480 nm, were taken every 15 min. Four replicates were run for each condition and the total assay was repeated once. After compensating for baseline measurements, fluorescence values were normalized to percentage of maximal response and plotted versus time.
